# Electroretinography and Gene Expression Measures Implicate Phototransduction and Metabolic Shifts in Chick Myopia and Hyperopia Models

**DOI:** 10.3390/life11060501

**Published:** 2021-05-29

**Authors:** Nina Riddell, Melanie J. Murphy, Sheila G. Crewther

**Affiliations:** School of Psychology and Public Health, La Trobe University, Melbourne, VIC 3086, Australia; M.Murphy@latrobe.edu.au (M.J.M.); s.crewther@latrobe.edu.au (S.G.C.)

**Keywords:** myopia, hyperopia, refractive error, microarray, gene expression, electroretinogram, ERG, GSEA

## Abstract

The Retinal Ion-Driven Fluid Efflux (RIDE) model theorizes that phototransduction-driven changes in trans-retinal ion and fluid transport underlie the development of myopia (short-sightedness). In support of this model, previous functional studies have identified the attenuation of outer retinal contributions to the global flash electroretinogram (gfERG) following weeks of myopia induction in chicks, while discovery-driven transcriptome studies have identified changes to the expression of ATP-driven ion transport and mitochondrial metabolism genes in the retina/RPE/choroid at the mid- to late-induction time-points. Less is known about the early time-points despite biometric analyses demonstrating changes in eye growth by 3 h in the chick lens defocus model. Thus, the present study compared gfERG and transcriptome profiles between 3 h and 3 days of negative lens-induced myopia and positive lens-induced hyperopia in chicks. Photoreceptor (a-wave and d-wave) and bipolar (b-wave and late-stage d-wave) cell responses were suppressed following negative lens-wear, particularly at the 3–4 h and 3-day time-points when active shifts in the rate of ocular growth were expected. Transcriptome measures revealed the up-regulation of oxidative phosphorylation genes following 6 h of negative lens-wear, concordant with previous reports at 2 days in this model. Signal transduction pathways, with core genes involved in glutamate and G-protein coupled receptor signalling, were down-regulated at 6 h. These findings contribute to a growing body of evidence for the dysregulation of phototransduction and mitochondrial metabolism in animal models of myopia.

## 1. Introduction

Myopia (short-sightedness) and hyperopia (long-sightedness) occur when the eye is too long or short, respectively, for its refractive power. Approximately 34% of the worldwide population are myopic, with prevalence expected to reach 50% by 2050 [1,2]. Myopia significantly increases the risk of vision loss from degenerative secondary disorders [3,4,5], making it a priority to understand the mechanisms underpinning ocular growth regulation.

Ocular growth has been shown to be controlled locally by the retina in a process that is dependent on visual feedback [6]. However, the biological mechanisms involved in the retinal control of eye growth are unresolved, though several models exist postulating roles for various signalling molecules and cell types [7,8,9]. Among these, the Retinal Ion-Driven Fluid Efflux (RIDE) model theorizes that myopia results from visually driven changes in phototransduction that decrease ion-driven fluid transport from the vitreous, across the RPE, to the choroid [7,10,11,12,13,14]. Due to its location and barrier function, others have similarly postulated a role for the photoreceptor/RPE interface in the development of myopia [15,16].

Evidence for the RIDE model derives primarily from animal studies using hatchling chicks. Rearing animals with negatively powered defocusing lenses or opaque occluders increases the rate of eye growth leading to myopia, whereas rearing with positively powered lenses arrests growth and leads to hyperopia [17,18,19,20,21,22]. The chick has been one of the most widely used animal models as, among other benefits, its response to optical manipulations is rapid and reliable [23] and molecular changes in the chick retina/RPE show similarities to the genes implicated in human myopia [24]. Using high throughput transcriptomics and proteomics, we recently identified changes in the expression of genes and proteins involved in phototransduction and cellular metabolism (particularly mitochondrial metabolism) as key molecular features of the retina/RPE response during the first 6 h of refractive error induction and recovery in chicks [25,26]. Most notably, photoreceptor proteins involved in the generation of the electroretinogram response were negatively correlated with refraction across negative and positive lens groups at this early time-point [26]. Furthermore, changes in the expression of ion transporters, including Na/K/ATPase and other ATP-powered pumps, were evident at later time-points, consistent with the expected link between cell activity, ion transport and associated metabolic needs during periods of altered eye growth [25,26,27,28,29]. Concurrent changes in the expression of immune (particularly complement-coagulation) and oxidative stress genes and proteins previously linked to maculopathy and choroidal neovasculatization suggests that downstream signalling cascades during periods of altered growth may combine with structural stress to predispose secondary pathology with age in high myopia [26,27,29,30,31].

The functional activity of outer retinal cells can be assessed using global flash electroretinograms (gfERGs), which measure the series of current loops that redistribute ions within the extracellular spaces of the retina following light-driven photoreceptor and bipolar cell polarity changes [32,33]. Previous studies have demonstrated that aspects of the gfERG waveform are suppressed following weeks of negative lens-wear and occlusion in chicks [14,34,35,36]. However, it is now known that refractive changes are initiated rapidly, with axial growth changes evident following 3 h of lens-wear [37,38], necessitating further functional studies within the first hours–days. Using multifocal ERG, Schmid et al. [39] demonstrated that just 2 h of occlusion in chicks disrupted the normal development of the ERG waveform. To our knowledge, gfERG responses in the positive and negative defocus model have not yet been examined during the initial phase of lens-wear. Thus, the primary aim of this study was to profile outer retinal function in the chick using gfERGs spanning 3–4 h to 3 days induction. We hypothesized that phototransduction changes would be evident at early time-points, consistent with the RIDE model and our previous proteomic findings in lens-induced myopia and hyperopia models [26].

The secondary aim of this study was to leverage existing transcriptome profiling to assist in building a systems-level understanding combining functional and gene expression measures in the chick defocus model. In a previous study, Stone et al. employed Affymetrix microarrays to profile the chick retina/RPE following 6 h and 3 days of positive and negative lens-wear [40]. Gene-level statistics implicated thousands of transcripts during lens-induced myopia induction, but only six transcripts under hyperopia induction conditions. Due to the single-gene focus of the original analysis, it remains unclear whether similar gene pathways are implicated at early time-points in the lens myopia and hyperopia models, as reported in our previous 6 h proteomics [26,28], or in transcriptome measures at later time-points [29,30]. Thus, we re-analysed Stone et al.’s data using the Gene Set Enrichment Analysis (GSEA) algorithm. Pathway analyses such as GSEA assist with interpretability issues when thousands of individual genes are implicated. As GSEA does not apply an arbitrary threshold for differential gene expression, this approach can also identify expression responses that are too subtle to detect at the single gene level [41,42], making it ideal for use with a dataset such as Stone et al.’s where both extremes are represented.

## 2. Materials and Methods

### 2.1. Electroretinogram of Retinal Function

#### 2.1.1. Animals and Rearing

Forty-eight male chicks (Leghorn/New Hampshire) were raised under a 12 h day/night light cycle from post-hatch day 1. Five hours into the light cycle on day 5, chicks were assigned to a lens condition (+10 dioptres; −10 dioptres; or No Lens), and lenses (8.1 mm polymethyl methacrylate contacts) were attached to the peri ocular feathers of the right eye. All procedures were conducted in accordance with the protocols approved by the La Trobe University Animal Ethics Committee and adhere to the ARVO Statement for the use of Animals in Ophthalmic and Vision Research.

#### 2.1.2. Electroretinograms

Following either 3–4 h, or 1, 2 or 3 days of lens-wear, four chicks per lens-group were anaesthetised (ketamine, 45 mg/kg; xylazine, 4.5 mg/kg i.m.) and retinal function was assessed using gfERG. Defocusing lenses remained in place during these ERG recordings in order to replicate the experimental differences between the chicks profiled using microarray (i.e., lens vs. no lens) and to prevent any functional recovery [37]. ERGs were recorded with an intravitreal electrode (Ag/AgCl), inserted into the right eye via a catheter placement unit, and a scleral reference. Chicks were dark adapted for 20 min prior to recordings. This timeframe was deemed sufficient as dark adaptation during the day in chicks levels off far quicker than at night due to a lack of rod contributions [43]. Consistent with this, research examining the effects of dark adaption in chicks across 1–40 min found no significant effect on a-wave or b-wave amplitude [44]. Following dark-adaptation, the retina was stimulated with a square-wave 500 ms onset, 2000 ms offset light flash (peak luminance of 50 cd/m^2^) generated by a 150 mm Ganzfeld stimulator. Potentials were recorded via a Powerlab (ADI, Sydney, Australia), and at least 80 responses were averaged from each chick.

#### 2.1.3. Data Analysis

**Figure 1:**
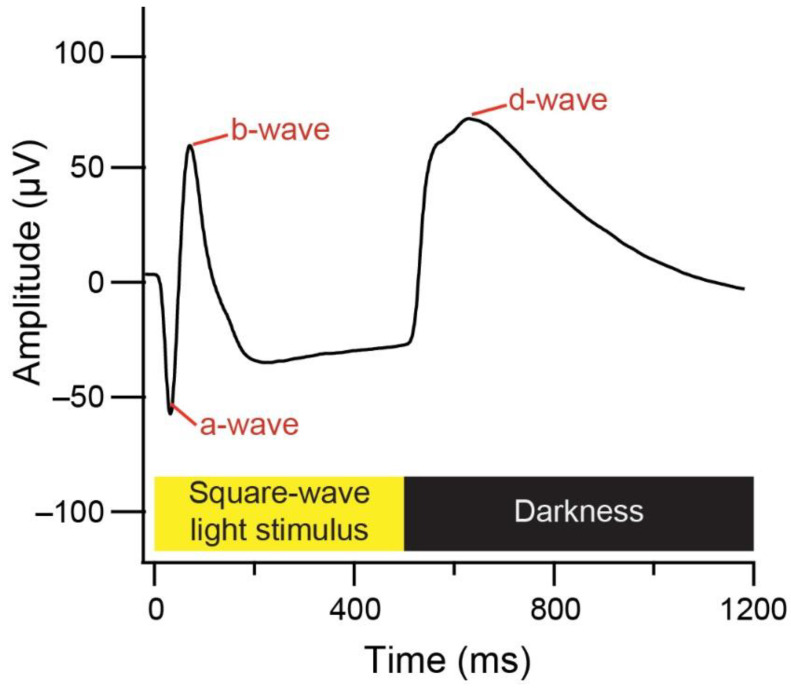
Example chick ERG response with the a-wave, b-wave, d-wave and stimulus onset/offset labelled.

ERG waves were imported into IGOR Pro where grand mean averages and dynamic standard error bars were calculated for each condition. Mean a-wave, b-wave, and d-wave amplitude and implicit time were extracted for each animal (see Figure 1 for example response). Wave amplitude was defined as the distance from peak to trough, while implicit time was defined as the time between the flash onset and the wave peak. Two-way ANOVAs were used to assess the effects of lens-wear on wave amplitudes and implicit times. Levene’s test was significant for a-wave amplitude at 3 h (*F* (2, 9) = 13.762, *p* = 0.002), d-wave amplitude at 72 h (*F* (2, 9) = 7.672, *p* = 0.011), and b-wave implicit time at 48 h (*F* (2, 9) = 12.667, *p* = 0.002). No adjustments were made to the analysis, as the *F* test is robust to such variance heterogeneity, provided group sizes are equal (as in the present study) [45]. The assumption of normality was met in all cases (Shapiro-Wilk *p* > 0.05 or z-scores < ±3.29 [46]).

### 2.2. Microarray Measures of Retina/RPE Gene Expression

#### 2.2.1. Data Pre-Processing

Stone et al.’s [40] microarray dataset was obtained from the GEO Database (GSE24641). A detailed description of the methods used to generate the dataset can be found in the original paper [40]. Briefly, in this previous study, chicks wore unilateral +15D or −15D lenses for 6 h or 3 days, with fellow no lens eyes used as within-subject controls. Retina/RPE tissue was collected from the experimental and fellow eyes of 6 chicks per group, and RNA was isolated from the samples using TRIzol reagent. RNA quantity and integrity were assessed using a spectrophotometer (ND-1000 NanoDrop Technologies, Wilmington, USA) and Bioanalyzer (Agilent Technologies, Santa Clara, USA) instruments. Gene expression was then profiled using Affymetrix Chicken Genome Arrays, according to the manufacturer’s instructions. Table 1 compares the rearing conditions used in the microarray and electrophysiology studies.

Raw microarray CEL files were pre-processed using the affy package (v1.52.0) implementation of robust multi-array analysis (RMA) [47,48], and probe annotations were updated to Affymetrix release 36.

#### 2.2.2. Gene Set Enrichment and Leading Edge Subset Analysis

After pre-processing, log-intensity values were imported into the java GSEA desktop application (v2.2.3) [49] to investigate the expression of Kyoto Encyclopaedia of Genes and Genomes (KEGG) [50] gene sets from the Molecular Signatures Database (mSigDB) [51]. GSEA was conducted with 1000 gene set permutations using the signal-to-noise metric. This metric uses the difference of class means scaled by the standard deviation to create a list of ranked genes. The analysis then determines whether genes from each pathway are randomly distributed through the ranked gene list, or primarily found at the top or bottom [41,49]. A false discovery rate (FDR) cut-off of 0.25 was chosen, as recommended by the Broad Institute when using the more stringent phenotype permutation option [41,52]. These parameters were used to make three comparisons at each time-point: negative lens relative to fellow no lens, positive lens relative to fellow no lens, and negative lens relative to positive lens.

Following the GSEA of KEGG pathways, leading edge subset analysis was employed to identify transcripts that were core contributors to the enriched signal of implicated gene sets (i.e., the core genes responsible for the gene set being designated as up- or down-regulated). This analysis lists genes appearing in the ranked gene set list at or before the point when the running sum reached its maximum deviation from zero [41].

## 3. Results

### 3.1. Electroretinography

**Figure 2:**
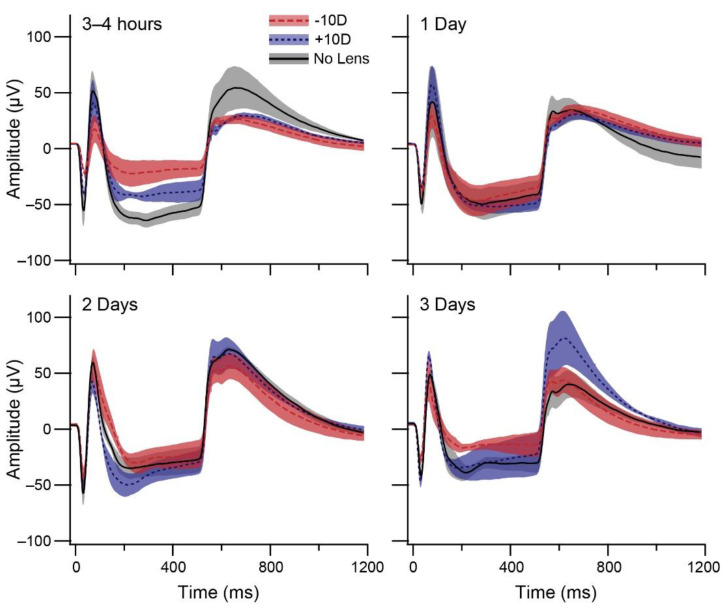
Graphs show mean ERG responses (±SE) to a square-wave light flash during refractive error induction. Chicks wore positive or negative lenses, or no lens, for 3–4 h, 1 day, 2 days, or 3 days. Where applicable, lenses remained in place during ERG recordings. Each waveform is the grand mean of >80 potential responses to a square-wave 0.5 s light ON, 2 s light OFF Ganzfeld stimulus from four chicks. Standard errors of the waves are indicated by the shaded regions surrounding the mean.

**Figure 3:**
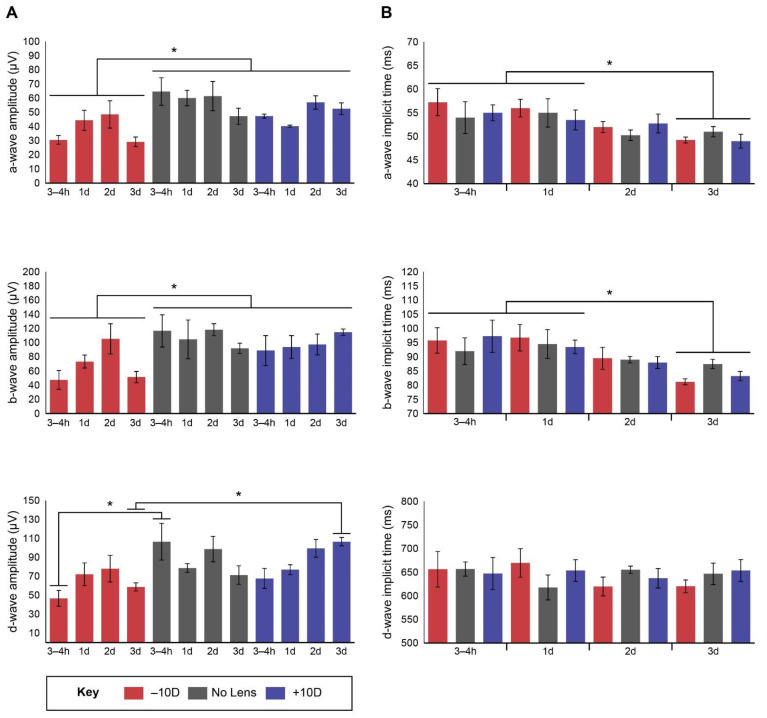
Bar graphs showing mean (±SE) ERG a-wave, b-wave and d-wave (**A**) amplitudes and (**B**) implicit times. Wave amplitude was defined as the distance from peak to trough, while implicit time was defined as the time between the flash onset and the wave peak (see Figure 1 for example). Statistically significant differences are indicated with an asterisk.

Figure 2 illustrates the retina’s functional response to light onset and offset across the refractive error induction period. Photoreceptor (a- and d-wave) and bipolar (b- and d-wave) cell responses appeared to be suppressed following negative lens-wear, particularly at 3–4 h. Two-way ANOVAs were conducted that examined the effects of lens-wear and induction timepoint on the amplitude (Figure 3A) and implicit time (Figure 3B) of the a-wave, b-wave and d-wave. There was a significant main effect of lens-wear on a-wave (*F* (2, 36) = 10.464, *p* < 001) and b-wave (*F* (2, 36) = 6.216, *p* = 0.005) amplitude. Post hoc tests revealed that a-wave and b-wave amplitude were significantly lower in the negative lens group relative to the no lens (a-wave Tukey HSD *p* < 001; b-wave Tukey HSD *p* = 0.005) and positive lens (a-wave Tukey HSD *p* = 0.043; b-wave Tukey HSD *p* = 0.037) groups (Figure 3A). There was a significant interaction between the effects of lens-wear and time on d-wave amplitude (*F* (6, 36) = 2.686, *p* = 0.029), such that d-wave amplitude was lower in the negative lens group relative to the no lens group at 3–4 h (*p* = 0.030) and the positive lens group at 3 days (*p* = 0.002; Figure 3A).

Lens-wear did not significantly affect the implicit time of the a-, b- or d-wave peaks. However, there was a main effect of induction timepoint on a-wave (*F* (3, 36) = 5.181, *p* = 0.004) and b-wave (*F* (3, 36) = 6.540, *p* = 0.001) implicit time, with lower implicit time at the 3-day timepoint relative to 3–4 h (a-wave Tukey HSD *p* = 0.009; b-wave Tukey HSD *p* = 0.003) and 1 day (a-wave Tukey HSD *p* = 0.021; b-wave Tukey HSD *p* = 0.004; Figure 3B). There were no significant main effects of lens-wear or timepoint, or interactions between lens-wear and timepoint, on the d-wave implicit time.

In order to retain the visual conditions present during rearing, all ERGs were recorded with the defocusing lenses still in place. Thus, the partial occlusion of the peripheral visual field by the surrounding Velcro ring may have contributed to response attenuation in the negative lens group. However, these conditions cannot fully account for the measured electrophysiological changes as differences remain between positive and negative groups.

### 3.2. Gene Pathway Enrichment

**Figure 4:**
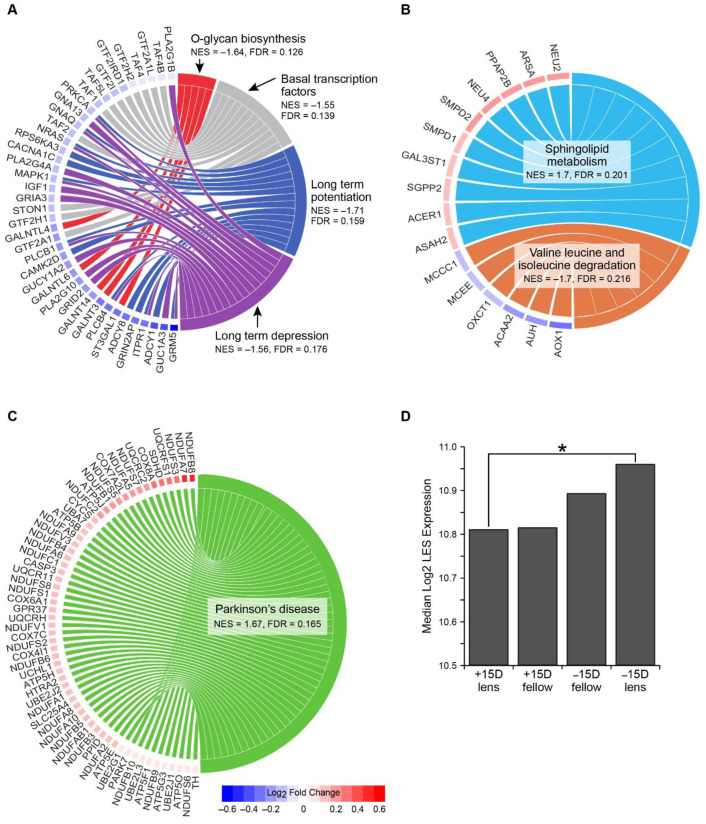
Gene contributions to KEGG pathway enrichment following 6 h of lens-wear. Chord diagrams show enriched KEGG pathways and their associated core genes in (**A**) the 6 h negative lens group relative to fellow eye controls, (**B**) the 6 h positive lens group relative to fellow eye controls, and (**C**) the 6 h negative relative to the positive lens group. Core genes are listed around the left half of each diagram (ordered from high to low log_2_ fold change). These core genes are defined as those appearing in the GSEA ranked list at or before the point when the enrichment score for the pathway reached its maximum deviation from zero (termed the leading edge subset; LES). The right half of each chord diagram shows the enriched pathways. Lines connecting the two halves indicate gene membership within a pathway’s LES. (**D**) Graph showing the median log_2_ expression of core genes from the Parkinson’s disease pathway within each condition at 6 h. Asterisks indicates groups showing a significant difference in the expression of ‘Parkinson’s disease’ genes in the GSEA analysis.

The re-analysis of Stone et al.’s microarray dataset identified seven enriched KEGG pathways at 6 h (Table 2). No pathways were enriched at 3 days. In keeping with functional ERG measures, two signal transduction pathways (long-term potentiation and long-term depression) were down-regulated following 6 h of negative lens-wear (Figure 4A). There was considerable overlap in the core genes contributing to the under-enrichment of these pathways, which included sub-units of ionotropic and metabotropic glutamate receptors (GRIN2A, GRIA3, GRID2, GRM5), and genes encoding G-proteins (GNA13, GNAQ), phospholipases (PLCB1, PLCB4, PLA2G1B, PLA2G4A), and adenylate cyclases (ADCY1, ADCY8) important for G-protein coupled receptor signalling. The basal transcription factor pathway was also down-regulated following 6 h of myopia induction. Core genes in this pathway included those encoding sub-units of TFIIA (GTF2A1, GTF2A1L), TFIID (TAF1, TAF2, TAF4, TAF4B), and TFIIH (GTF2H1, GTF2H2) in the RNA polymerase II pre-initiation complex. Concurrent with the expression changes in this transcription pathway, the O-glycan biosynthesis post-translational modification pathway was down-regulated. The core genes contributing to this down-regulation primarily encoded members of the polypeptide N-acetylgalactosaminyltransferase enzyme family. Following 6 h of positive lens-wear, the valine leucine and isoleucine degradation pathway was down-regulated, and the sphingolipid metabolism pathway was up-regulated (Figure 4B). Finally, the Parkinson’s disease pathway was enriched in the negative relative to positive lens-group at 6 h (Figure 4C). The core genes in this pathway were primarily involved in mitochondrial energy metabolism. A further comparison of core genes within the Parkinson’s pathway across all lens groups indicated that fellow eyes showed similar expression profiles as experimental eyes (i.e., the median level of core gene expression in negative lens eyes and their fellow no lens controls was higher than that in positive lens eyes and their fellows; Figure 4D).

## 4. Discussion

This study compared functional gfERG and microarray transcriptome measures across 3 days of lens-induced myopia and hyperopia induction in chicks. As hypothesized, electrophysiology revealed the attenuation of the photoreceptor and bipolar cell response to flash onset and offset following negative lens-wear. The largest mean differences in a-wave, b-wave and d-wave amplitude occurred at 3–4 h and 3 days when shifts in the rate of ocular growth are expected (i.e., the initiation of growth changes during the first hours of lens-wear and when refractive compensation is reached at 3 days [29]). Transcriptome measures replicated and extended the findings of our previous studies [25,27,29] to show that increases in the expression of oxidative phosphorylation genes occur as early as 6 h in the chick myopia model, accompanied by the down-regulation of signal transduction pathways that include genes important for photoreceptor function.

Although the photoreceptor/RPE interface is theorized to be involved in ocular growth regulation [7,15,16], measuring the functioning of these cells during refractive error induction via flash ERG is complicated because axial length changes are expected to affect retinal illumination, the distance between the electrode and the retina, and retinal cell density [32]. Consequently, although a number of studies have reported reduced ERG amplitudes in human myopes [53,54,55,56,57] and in the chick model of prolonged high myopia [14,34,35], these decreases have often been attributed to recording artefacts, retinal thinning, and pathological retinal degeneration. The present study expands on these earlier investigations to demonstrate that ERG measures of photoreceptor (a-wave), ON-bipolar (b-wave) and photoreceptor/OFF-bipolar (d-wave) cell responses are attenuated following 3–4 h of negative lens-wear (presumably prior to the onset of significant pathological axial growth, retinal thinning, or degeneration). Indeed, the absence of a significant effect of induction time on ERG wave amplitudes in the present study suggests that axial length artefacts may have a minimal effect on the gfERG in the small chick eye, consistent with previous multifocal ERG reports in humans [58]. We did identify a decrease in a-wave and b-wave implicit time with induction time independent of lens defocus, consistent with investigations of ERG maturation in humans [59,60] and chicks [61].

Our previous proteomic study demonstrated that photoreceptor proteins (particularly those involved in abnormal electroretinogram responses in humans) were negatively correlated with refraction across negative and positive lens groups at 6 h [26]. Photoreceptor-specific pathways were not implicated in the present study, however, two signal transduction pathways (long-term potentiation and long-term depression) were down-regulated following 6 h of negative lens-wear. The core genes within these pathways suggest a particular role for glutamate and G-protein coupled receptor signalling, consistent with Stone et al.’s original analysis [40]. Phospholipases, important for rod outer segment membrane regeneration and the subsequent shaping of the photo-response, were also implicated [62,63]. Previous studies have similarly found evidence in lens and occlusion myopia models for ERG response attenuation [14,34,35,36,64] and changes in the expression of genes and proteins involved in outer retinal functioning (particularly phototransduction) [25,26,29].

In darkness, photoreceptors consume large amounts of ATP to power the ion pumps that maintain the membrane potential; exposure to light diminishes this ATP consumption [65]. Accordingly, the attenuation of the photoreceptor light response during negative lens-wear in the present study would be expected to affect the metabolic needs of the outer retina. In addition to ATP-driven ion transport, metabolic flux during refractive error induction is likely to be affected by changes to nutrient supply from the choroidal vasculature [66,67] and altered metabolic pressures for cell growth and proliferation [68]. In accordance with this, our previous RNA-seq study identified the up-regulation of the KEGG Parkinson’s disease pathway (with the core genes primarily related to mitochondrial oxidative phosphorylation) following 2 days of lens-induced myopia relative to age-matched no lens and positive lens groups. Here, we show that the relative enrichment of this pathway is negative relative to the positive lens groups which occurs as early as 6 h in chicks. However, contrary to our earlier results with separate no lens control animals, fellow no lens eyes in the present study displayed similar expression patterns to the contralateral experimental eye. This suggests that a factor affecting both eyes (such as blood supply changes [67]) could drive the shifts in mitochondrial metabolism gene and protein expression observed here and elsewhere [25,26,27,29,30,69].

Shifts in the expression of sphingolipid, and valine leucine and isoleucine degradation pathways following 6 h of positive lens-wear in the present study are also concordant with our RNA-sequencing findings at later time-points in the defocus model. Previously, we identified the down-regulation of a range of lipid-related genes following 1 day of positive lens-wear, including genes within sphingolipid metabolism and fatty acid metabolism pathways [29]. In the present study, the sphingolipid metabolism and branched chain amino acid (BCAA) degradation pathways were up-regulated and down-regulated, respectively, following 6 h of positive lens-wear. These pathways are involved in a diverse range of biological processes, including the regulation of cellular energy metabolism [70,71,72,73,74]. Notably, BCAA catabolism products are incorporated into the tricarboxylic acid cycle (TCA) cycle resulting in increased fatty acid oxidation (possibly via an inhibition of pyruvate dehydrogenase that shifts the cellular energy preference from carbohydrate to lipid) [70,71]. This suggests that shifts in the BCAA degradation pathway during early hyperopia induction could precipitate the down-regulation of fatty acid metabolism genes observed in our previous study [29].

The two final gene pathways implicated in the present study were basal transcription factors and O-glycan biosynthesis; both were down-regulated following 6 h of negative lens-wear. The core genes in the transcription factor pathway primarily encoded subunits of the RNA polymerase II pre-initiation complex, which may help to explain why the pattern of differential expression identified in Stone et al.’s original analysis of the data [40] was overwhelmingly characterized by down-regulation (1252 probes were down-regulated compared to 139 up-regulated). The core genes in the O-glycosylation pathway primarily encoded enzymes involved in GalNAc-type (also known as mucin-type) O-glycosylation of proteins. This family of enzymes are thought to influence ECM composition via a number of pathways previously associated with myopia (including integrin, fibroblast growth factor [75], and TGF-β/BMP signalling, e.g., [76,77]). Interestingly, the genetic defects of protein glycosylation have been linked to myopia in humans, as well as the suppression of the scotopic ERG [78].

Although a range of pathways were implicated in the 6 h data, no significant pathway enrichments were identified following 3 days of lens-wear. This suggests that coherent shifts in KEGG pathway expression may decline as an eye approaches refractive compensation and growth homeostasis. It is unlikely that this finding reflects a lack of sensitivity in the methods used; GSEA is a highly sensitive pathway analysis technique [79] and we employed a relatively high FDR threshold of 0.25 (as recommended for discovery-driven projects using phenotype permutation [41,52]). Additionally, a similar pattern of decreasing pathway enrichments as refractive compensation progressed was observed in our RNA-seq GSEA study [29]. It must be noted, however, that the scope of both analyses was limited to pathways represented in the KEGG database, which covers a wide range of categories (including metabolism, genetic information processing, environmental information processing, and cellular processes), but has a particular focus on metabolic pathways. KEGG was chosen for use as the data are manually curated by experts and there is a low level of pathway redundancy (relative to other databases like Reactome) [80,81]. However, given the lack of pathway enrichments following 3 days of negative lens-wear (when Stone et al.’s previous analysis identified >1200 differentially expressed transcripts [40]), we performed an additional GSEA of these data using the mSigDB ‘C2_all’ GMT file (which encompasses all curated gene set databases, including Reactome and BioCarta). No significant enrichments were identified in this expanded analysis, supporting the assertion that coherent shifts in whole-pathway expression were limited at 3 days.

## 5. Limitations

The ERG protocol employed in this study was basic, involving a single intensity and duration of flash stimulus. Extended ERG protocols (e.g. [14,82]) would allow for a more detailed assessment of outer retinal functioning. To complement the ERG data, gene expression in the retina/RPE was examined using a previously published exploratory microarray dataset. DNA microarray technology provides a cost-effective approach for examining transcriptome-wide expression but has a number of limitations including a small dynamic range and lack of sensitivity (particularly when measuring low abundance transcripts) [83]. Thus it is important to note that the gene expression findings reported here are consistent with reports using newer proteomic [26] and RNA-sequencing [25,29] technologies. Further work is now needed to follow up the present findings using a more comprehensive electrophysiological analysis, and targeted molecular and histological examination of photoreceptor and metabolic functioning in the retina. Our results suggest that the initial hours of myopia induction, and the period when refractive compensation is achieved, are important timepoints for such further studies in the chick optical defocus model.

## 6. Conclusions

Our novel gfERG data and re-analysis of Stone et al.’s dataset provide evidence that changes to phototransduction and retinal metabolism occur within the first 6 h of refractive compensation to defocusing lenses in chicks. These findings contribute to a growing body of electrophysiological [14], structural [13,84] and molecular [25,26,27,29,69,85,86,87,88] evidence for altered phototransduction and consequential associated shifts in retinal mitochondrial metabolism in animal myopia models, consistent with the RIDE model of myopia development [7,11,12]. A recent meta-analysis of genome-wide association studies (GWAS) has similarly identified the phototransduction cascade as the most significant process associated with refractive errors in humans [89]. Given this evidence, future research examining environmental or pharmacological interventions targeting photoreceptor activity or associated ion-driven fluid efflux across the retina is warranted.

## Figures and Tables

**Table 1:** A comparison of chick rearing methods for the Stone et al. microarray [40] and Riddell et al. electrophysiology data.

Method	Microarray Data [40]	Riddell et al. Electrophysiology Data
Breed	White Leghorn	White Leghorn/New Hampshire
Refractive error model	Optical defocus	Optical defocus
Lens power	+15D; −15D	+10D; −10D
Age lens applied	8 days	5 days
Hours into light phase lens applied	1 h	5 h
Induction time-points	6 h, 3 days	3–4 h and 1, 2 and 3 days

**Table 2:** Enriched KEGG pathways following 6 h of lens-wear. NES = normalized enrichment score; Tags = the percentage of gene hits before (for a positive value ES) or after (for a negative value ES) the peak in the running enrichment score. This gives an indication of the percentage of genes in the pathway contributing to the enrichment score. List = the percentage of genes in the ranked list before (for a positive value ES) or after (for a negative value ES) the peak in the running enrichment score. This gives an indication of where in the list the enrichment score was attained. Signal = the enrichment signal strength that combines the two previous statistics.

Condition	KEGG Pathway	NES	FDR	Tags	List	Signal
Negative lens vs. fellow no lens at 6 h	O-glycan biosynthesis	−1.64	0.126	19%	7%	21%
	Basal transcription factors	−1.55	0.139	44%	21%	57%
	Long-term potentiation	−1.71	0.159	28%	9%	31%
	Long-term depression	−1.56	0.176	31%	13%	35%
Positive lens vs. fellow no lens at 6 h	Sphingolipid metabolism	1.70	0.201	30%	10%	34%
	Valine, leucine, and isoleucine degradation	−1.70	0.216	15%	10%	17%
Negative lens vs. positive lens at 6 h	Parkinson’s disease	1.67	0.165	66%	42%	114%

## Data Availability

The microarray data analysed in this paper are available in the Gene Expression Omnibus (GEO) database under accession number GSE24641. Raw ERG waves are available from the authors upon request.

## References

[1] Holden B.A., Fricke T.R., Wilson D.A., Jong M., Naidoo K.S., Sankaridurg P., Wong T.Y., Naduvilath T.J., Resnikoff S. (2016). Global Prevalence of Myopia and High Myopia and Temporal Trends from 2000 through 2050. Ophthalmology.

[2] Hashemi H., Fotouhi A., Yekta A., Pakzad R., Ostadimoghaddam H., Khabazkhoob M. (2018). Global and regional estimates of prevalence of refractive errors: Systematic review and meta-analysis. J. Curr. Ophthalmol..

[3] Verhoeven V.J., Wong K.T., Buitendijk G.H., Hofman A., Vingerling J.R., Klaver C.C. (2015). Visual consequences of refractive errors in the general population. Ophthalmology.

[4] Flitcroft D.I. (2012). The complex interactions of retinal, optical and environmental factors in myopia aetiology. Prog. Retin. Eye Res..

[5] Saw S.M., Gazzard G., Shih-Yen E.C., Chua W.H. (2005). Myopia and associated pathological complications. Ophthalmic Physiol. Opt..

[6] Troilo D., Smith E.L., Nickla D.L., Ashby R., Tkatchenko A.V., Ostrin L.A., Gawne T.J., Pardue M.T., Summers J.A., Kee C.S. (2019). IMI—Report on Experimental Models of Emmetropization and Myopia. Investig. Ophthalmol. Vis. Sci..

[7] Crewther D.P. (2000). The role of photoreceptors in the control of refractive state. Prog. Retin. Eye Res..

[8] Chakraborty R., Pardue M.T. (2015). Molecular and biochemical aspects of the retina on refraction. Prog. Mol. Biol. Transl. Sci..

[9] Wallman J., Winawer J. (2004). Homeostasis of eye growth and the question of myopia. Neuron.

[10] Liang H., Crewther S., Crewther D., Dickson M., Junghans B. (1998). X-ray microanalysis of the chick choroid and retina in experimental myopia. Investig. Ophthalmol. Vis. Sci..

[11] Liang H., Crewther S.G., Crewther D.P., Junghans B.M. (2004). Structural and elemental evidence for edema in the retina, retinal pigment epithelium, and choroid during recovery from experimentally induced myopia. Investig. Ophthalmol. Vis. Sci..

[12] Crewther S.G., Liang H., Junghans B.M., Crewther D.P. (2006). Ionic control of ocular growth and refractive change. Proc. Natl. Acad. Sci. USA.

[13] Liang H., Crewther D., Gillard Crewther S., Barila A. (1995). A role for photoreceptor outer segments in the induction of deprivation myopia. Vision Res..

[14] Westbrook A.M., Crewther D.P., Crewther S.G. (1999). Cone receptor sensitivity is altered in form deprivation myopia in the chicken. Optom. Vis. Sci..

[15] Rymer J., Wildsoet C.F. (2005). The role of the retinal pigment epithelium in eye growth regulation and myopia: A review. Vis. Neurosci..

[16] Zhang Y., Wildsoet C.F. (2015). RPE and choroid mechanisms underlying ocular growth and myopia. Prog. Mol. Biol. Transl. Sci..

[17] Schaeffel F., Glasser A., Howland H.C. (1988). Accommodation, refractive error and eye growth in chickens. Vis. Res..

[18] Crewther S.G., Nathan J., Kiely P.M., Brennan N.A., Crewther D.P. (1988). The Effect of Defocusing Contact-Lenses on Refraction in Cynomolgus Monkeys. Clin. Vis. Sci..

[19] Nathan J., Crewther S.G., Crewther D.P., Kiely P.M. (1984). Effects of Retinal Image Degradation on Ocular Growth in Cats. Investig. Ophthalmol. Vis. Sci..

[20] Stone R.A., Lin T., Laties A.M., Iuvone P.M. (1989). Retinal dopamine and form-deprivation myopia. Proc. Natl. Acad. Sci. USA.

[21] Wiesel T.N., Raviola E. (1977). Myopia and eye enlargement after neonatal lid fusion in monkeys. Nature.

[22] Wallman J., Turkel J., Trachtman J. (1978). Extreme myopia produced by modest change in early visual experience. Science.

[23] Schaeffel F., Feldkaemper M. (2015). Animal models in myopia research. Clin. Exp. Optom..

[24] Riddell N., Crewther S.G. (2017). Integrated Comparison of GWAS, Transcriptome, and Proteomics Studies Highlights Similarities in the Biological Basis of Animal and Human Myopia. Investig. Ophthalmol. Vis. Sci..

[25] Vocale L.G., Crewther S., Riddell N., Hall N.E., Murphy M., Crewther D. (2021). RNA-seq and GSEA identifies suppression of ligand-gated chloride efflux channels as the major gene pathway contributing to form deprivation myopia. Sci. Rep..

[26] Riddell N., Faou P., Murphy M., Giummarra L., Downs R.A., Rajapaksha H., Crewther S.G. (2017). The retina/RPE proteome in chick myopia and hyperopia models: Commonalities with inherited and age-related ocular pathologies. Mol. Vis..

[27] Giummarra L., Crewther S.G., Riddell N., Murphy M.J., Crewther D.P. (2018). Pathway analysis identifies altered mitochondrial metabolism, neurotransmission, structural pathways and complement cascade in retina/RPE/ choroid in chick model of form-deprivation myopia. PeerJ.

[28] Riddell N., Faou P., Crewther S.G. (2018). Short term optical defocus perturbs normal developmental shifts in retina/RPE protein abundance. BMC Dev. Biol..

[29] Riddell N., Giummarra L., Hall N., Crewther S. (2016). Bidirectional Expression of Metabolic, Structural, and Immune Pathways in Early Myopia and Hyperopia. Front. Neurosci..

[30] Riddell N., Crewther S.G. (2017). Novel evidence for complement system activation in chick myopia and hyperopia models: A meta-analysis of transcriptome datasets. Sci. Rep..

[31] Francisco B.-M., Salvador M., Amparo N. (2015). Oxidative Stress in Myopia. Oxid. Med. Cell. Longev..

[32] Heckenlively J.R., Arden G.B. (2006). Principles and Practice of Clinical Electrophysiology of Vision.

[33] Robson J.G., Frishman L.J. (1998). Dissecting the dark-adapted electroretinogram. Doc. Ophthalmol..

[34] Fujikado T., Kawasaki Y., Suzuki A., Ohmi G., Tano Y. (1997). Retinal function with lens-induced myopia compared with form-deprivation myopia in chicks. Graefes Arch. Clin. Exp. Ophthalmol..

[35] Fujikado T., Hosohata J., Omoto T. (1996). ERG of form deprivation myopia and drug induced ametropia in chicks. Curr. Eye Res..

[36] Hodos W., Fitzke F.W., Hayes B.P., Holden A.L. (1985). Experimental myopia in chicks: Ocular refraction by electroretinography. Investig. Ophthalmol. Vis. Sci..

[37] Zhu X.Y., Park T.W., Winawer J., Wallman J. (2005). In a matter of minutes, the eye can know which way to grow. Investig. Ophthalmol. Vis. Sci..

[38] Kee C., Marzani D., Wallman J. (2001). Differences in time course and visual requirements of ocular responses to lenses and diffusers. Investig. Ophthalmol. Vis. Sci..

[39] Schmid K.L., Rayner C.L., Brown B. (2013). Hemi-field and full-field form-deprivation induce timing changes in multifocal ERG responses in chick. Ophthalmic Physiol. Opt..

[40] Stone R.A., McGlinn A.M., Baldwin D.A., Tobias J.W., Iuvone P.M., Khurana T.S. (2011). Image defocus and altered retinal gene expression in chick: Clues to the pathogenesis of ametropia. Investig. Ophthalmol. Vis. Sci..

[41] Subramanian A., Tamayo P., Mootha V.K., Mukherjee S., Ebert B.L., Gillette M.A., Paulovich A., Pomeroy S.L., Golub T.R., Lander E.S. (2005). Gene set enrichment analysis: A knowledge-based approach for interpreting genome-wide expression profiles. Proc. Natl. Acad. Sci. USA.

[42] Manoli T., Gretz N., Grone H.J., Kenzelmann M., Eils R., Brors B. (2006). Group testing for pathway analysis improves comparability of different microarray datasets. Bioinformatics.

[43] Schaeffel F., Rohrer B., Lemmer T., Zrenner E. (1991). Diurnal control of rod function in the chicken. Vis. Neurosci..

[44] Ookawa T. (1971). Further studies on the ontogenetic development of the chick electroretinogram. Poult. Sci..

[45] Blanca M.J., Alarcon R., Arnau J., Bono R., Bendayan R. (2018). Effect of variance ratio on ANOVA robustness: Might 1.5 be the limit?. Behav. Res. Methods.

[46] Tabachnick B.G., Fidell L.S., Ullman J.B. (2007). Using Multivariate Statistics.

[47] Gautier L., Cope L., Bolstad B.M., Irizarry R.A. (2004). affy—Analysis of Affymetrix GeneChip data at the probe level. Bioinformatics.

[48] Irizarry R.A., Bolstad B.M., Collin F., Cope L.M., Hobbs B., Speed T.P. (2003). Summaries of Affymetrix GeneChip probe level data. Nucleic Acids Res..

[49] Subramanian A., Kuehn H., Gould J., Tamayo P., Mesirov J.P. (2007). GSEA-P: A desktop application for Gene Set Enrichment Analysis. Bioinformatics.

[50] Kanehisa M., Goto S. (2000). KEGG: Kyoto encyclopedia of genes and genomes. Nucleic Acids Res..

[51] Liberzon A., Subramanian A., Pinchback R., Thorvaldsdottir H., Tamayo P., Mesirov J.P. (2011). Molecular signatures database (MSigDB) 3.0. Bioinformatics.

[52] Gene Set Enrichment Analysis: GSEA User Guide. https://www.gsea-msigdb.org/gsea/doc/GSEAUserGuideFrame.html.

[53] Blach R.K., Jay B., Kolb H. (1966). Electrical activity of the eye in high myopia. Br. J. Ophthalmol..

[54] Kader M.A. (2012). Electrophysiological study of myopia. Saudi J. Ophthalmol..

[55] Malik S.R., Gupta A.K., Gupta P.C., Singh G. (1969). E.R.G. in myopia. J. All India Ophthalmol. Soc..

[56] Perlman I., Meyer E., Haim T., Zonis S. (1984). Retinal Function in High Refractive Error Assessed Electroretinographically. Br. J. Ophthalmol..

[57] Westall C.A., Dhaliwal H.S., Panton C.M., Sigesmun D., Levin A.V., Nischal K.K., Heon E. (2001). Values of electroretinogram responses according to axial length. Doc. Ophthalmol..

[58] Chen J.C., Brown B., Schmid K.L. (2006). Delayed mfERG responses in myopia. Vis. Res..

[59] Westall C.A., Panton C.M., Levin A.V. (1998). Time courses for maturation of electroretinogram responses from infancy to adulthood. Doc. Ophthalmol..

[60] Birch D.G., Anderson J.L. (1992). Standardized full-field electroretinography. Normal values and their variation with age. Arch. Ophthalmol..

[61] Ookawa T. (1971). The onset and development of the chick electroretinogram: The A- and B-waves. Poult. Sci..

[62] Jelsema C.L. (1987). Light activation of phospholipase A2 in rod outer segments of bovine retina and its modulation by GTP-binding proteins. J. Biol. Chem..

[63] Jastrzebska B., Debinski A., Filipek S., Palczewski K. (2011). Role of membrane integrity on G protein-coupled receptors: Rhodopsin stability and function. Prog. Lipid Res..

[64] Sieck E., Enzenauer R., Pedler M. Effect of Induced Refractive Error on Electroretinograms. Proceedings of the ARVO Conference.

[65] Hurley J.B., Lindsay K.J., Du J. (2015). Glucose, lactate, and shuttling of metabolites in vertebrate retinas. J. Neurosci. Res..

[66] Shih Y.-F., Fitzgerald M.E., Norton T.T., Gamlin P.D., Hodos W., Reiner A. (1993). Reduction in choroidal blood flow occurs in chicks wearing goggles that induce eye growth toward myopia. Curr. Eye Res..

[67] Jin N., Stjernschantz J. (2000). Regional blood flow in the myopic chick eye during and after form deprivation: A study with radioactively-labelled microspheres. Exp. Eye Res..

[68] Lunt S.Y., Vander Heiden M.G. (2011). Aerobic glycolysis: Meeting the metabolic requirements of cell proliferation. Annu. Rev. Cell Dev. Biol..

[69] Barathi V.A., Chaurasia S.S., Poidinger M., Koh S.K., Tian D., Ho C., Iuvone P.M., Beuerman R.W., Zhou L. (2014). Involvement of GABA transporters in atropine-treated myopic retina as revealed by iTRAQ quantitative proteomics. J. Proteome Res..

[70] Zhang S., Zeng X., Ren M., Mao X., Qiao S. (2017). Novel metabolic and physiological functions of branched chain amino acids: A review. J. Anim. Sci. Biotechnol..

[71] Kainulainen H., Hulmi J.J., Kujala U.M. (2013). Potential role of branched-chain amino acid catabolism in regulating fat oxidation. Exerc. Sport Sci. Rev..

[72] Zeidan Y.H., Hannun Y.A. (2007). Translational aspects of sphingolipid metabolism. Trends Mol. Med..

[73] Bikman B.T., Summers S.A. (2011). Ceramides as modulators of cellular and whole-body metabolism. J. Clin. Investig..

[74] Aguilera-Romero A., Gehin C., Riezman H. (2014). Sphingolipid homeostasis in the web of metabolic routes. Biochim. Biophys. Acta.

[75] Tian E., Hoffman M.P., Ten Hagen K.G. (2012). O-glycosylation modulates integrin and FGF signalling by influencing the secretion of basement membrane components. Nat. Commun..

[76] Herr P., Korniychuk G., Yamamoto Y., Grubisic K., Oelgeschlager M. (2008). Regulation of TGF-beta signalling by N-acetylgalactosaminyltransferase-like 1. Development.

[77] Fakhro K.A., Choi M., Ware S.M., Belmont J.W., Towbin J.A., Lifton R.P., Khokha M.K., Brueckner M. (2011). Rare copy number variations in congenital heart disease patients identify unique genes in left-right patterning. Proc. Natl. Acad. Sci. USA.

[78] Jensen H., Kjaergaard S., Klie F., Moller H.U. (2003). Ophthalmic manifestations of congenital disorder of glycosylation type 1a. Ophthalmic Genet..

[79] Bayerlova M., Jung K., Kramer F., Klemm F., Bleckmann A., Beissbarth T. (2015). Comparative study on gene set and pathway topology-based enrichment methods. BMC Bioinform..

[80] Bauer-Mehren A., Furlong L.I., Sanz F. (2009). Pathway databases and tools for their exploitation: Benefits, current limitations and challenges. Mol. Syst. Biol..

[81] Chowdhury S., Sarkar R.R. (2015). Comparison of human cell signaling pathway databases--evolution, drawbacks and challenges. Database (Oxford).

[82] Ahmad J.M., Ioshimoto G., Liber A., Ventura D. (2019). Scotopic ERG Protocols for Rabbits. Investig. Ophthalmol. Vis. Sci..

[83] Czechowski T., Bari R.P., Stitt M., Scheible W.R., Udvardi M.K. (2004). Real-time RT-PCR profiling of over 1400 Arabidopsis transcription factors: Unprecedented sensitivity reveals novel root- and shoot-specific genes. Plant J..

[84] Liang H., Crewther S.G., Crewther D.P., Pirie B. (1996). Morphology of the recovery from form deprivation myopia in the chick. Aust. N. Z. J. Ophthalmol..

[85] Wang B., Liu Y., Chen S., Wu Y., Lin S., Duan Y., Zheng K., Zhang L., Gu X., Hong W. (2017). A Novel Potentially Causative Variant of NDUFAF7 Revealed by Mutation Screening in a Chinese Family With Pathologic Myopia. Investig. Ophthalmol. Vis. Sci..

[86] Mo Y., He M.L., Yu J.Z., Xie X.J. (2021). Bioinformatics analysis of the gene expression profile of retinal pigmental epithelial cells based in single-cell RNA sequencing in myopic mice. Arch. Med. Sci..

[87] Bian J., Sze Y.H., Tse D.Y., To C.H., McFadden S.A., Lam C.S., Li K.K., Lam T.C. (2021). SWATH Based Quantitative Proteomics Reveals Significant Lipid Metabolism in Early Myopic Guinea Pig Retina. Int. J. Mol. Sci..

[88] Yang J., Reinach P.S., Zhang S., Pan M., Sun W., Liu B., Li F., Li X., Zhao A., Chen T. (2017). Changes in retinal metabolic profiles associated with form deprivation myopia development in guinea pigs. Sci. Rep..

[89] Tedja M.S., Wojciechowski R., Hysi P.G., Eriksson N., Furlotte N.A., Verhoeven V.J.M., Iglesias A.I., Meester-Smoor M.A., Tompson S.W., Fan Q. (2018). Genome-wide association meta-analysis highlights light-induced signaling as a driver for refractive error. Nat. Genet..

